# Pediatric Medulloblastoma – Update on Molecular Classification Driving Targeted Therapies

**DOI:** 10.3389/fonc.2014.00176

**Published:** 2014-07-22

**Authors:** Ruth-Mary DeSouza, Benjamin R. T. Jones, Stephen P. Lowis, Kathreena M. Kurian

**Affiliations:** ^1^Neurosurgery, King’s College Hospital London, London, UK; ^2^Royal Bolton Hospital, Bolton, UK; ^3^BMT/Oncology, University of Bristol, Bristol, UK; ^4^Brain Tumour Group, Institute of Clinical Neuroscience, University of Bristol, Bristol, UK

**Keywords:** pediatric, medulloblastoma, molecular, therapies, classification

## Abstract

As advances in the molecular and genetic profiling of pediatric medulloblastoma evolve, associations with prognosis and treatment are found (prognostic and predictive biomarkers) and research is directed at molecular therapies. Medulloblastoma typically affects young patients, where the implications of any treatment on the developing brain must be carefully considered. The aim of this article is to provide a clear comprehensible update on the role molecular profiling and subgroups in pediatric medulloblastoma as it is likely to contribute significantly toward prognostication. Knowledge of this classification is of particular interest because there are new molecular therapies targeting the Shh subgroup of medulloblastomas.

## Introduction

**Figure 1:**
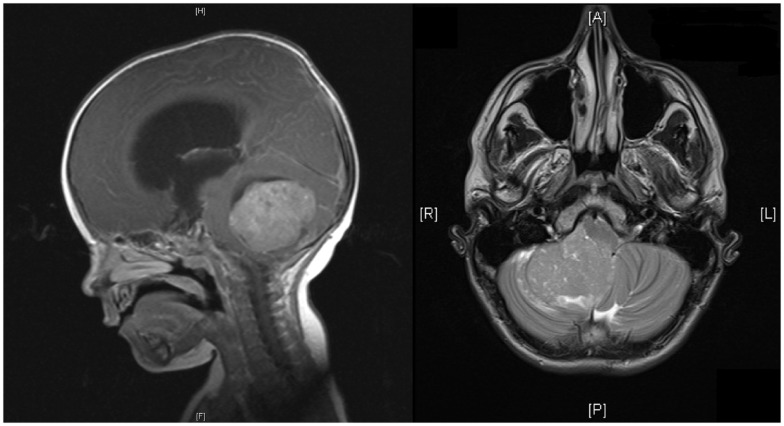
**MRI of head showing sagittal and horizontal views**. Sagittal view shows a midline posterior fossa medulloblastoma with intermediate signal intensity. There is an obstruction to the flow of CSF, marked hydrocephalus, and edema. Horizontal view shows a homogenous enhancing medulloblastoma arising from the right cerebellar hemisphere with displacement of the vermis.

Brain tumors are the most common solid malignancies in children, and among these medulloblastoma is the most frequent (1). The incidence of medulloblastoma is higher in males and higher in early childhood, with almost half occurring before the age of 5 (1). At least 75% of childhood medulloblastomas arise in the cerebellar vermis, and project into the fourth ventricle, with the remainder involving the cerebellar hemispheres (see Figure 1) (1). Spread may be via CSF and present in up to one-third of cases at presentation (2).

The clinical features of medulloblastoma, as with other posterior fossa pathology, can be difficult to detect initially in young children, sometimes leading to a delayed diagnosis (3). Symptoms include headache, general malaise, failure to feed, vomiting, clumsiness, and other presentations that mimic common and benign childhood pathologies seen in primary care (4). Typically, the treatment strategies for medulloblastoma are threefold: maximal safe resection (plus/minus CSF diversion), neuraxis radiotherapy, and chemotherapy (4).

Survival in children with medulloblastoma has improved over the last 20 years, and the quality of life in medulloblastoma survivors has been evaluated in terms of physical and non-physical (5). Physical impairments include neurological deficits, secondary malignancy, and endocrine dysfunction, whereas non-physical deficits include cognitive difficulties and psychological and social problems (6). The effect of these problems can be far reaching, affecting employment and family life (7–9). Many long-term sequelae are secondary to radiotherapy and one of the goals of modern therapy is to minimize or avoid radiotherapy (7–9).

## Clinical Classification

Medulloblastoma was classified clinically by Chang in 1969 based on the size and invasiveness of the tumor as determined intra-operatively and on the presence of metastases (10). The Chang system is no longer used, although elements of it form the current clinical risk stratification of medulloblastoma (10). Currently, medulloblastoma is classified clinically into high risk and standard (average) risk, which is summarized in Table 1. The factors contributing to this classification are solely clinical – age, metastases, and resection (3). Age is a key factor, which may reflect in part the aggressive natural history of tumors in the under-three age group and also reflect the limitations and side effects of therapy (3).

**Table 1:** **Established prognostic variables accepted by the North American Children’s Oncology Group (COG) and the SIOP (International Society of Pediatric Oncology) Group**.

Risk classification	Characteristics
Standard-risk tumor	≥3 years of age without evidence of metastatic spread and having ≤1.5 cm^2^ (maximum cross- sectional area) of residual disease after surgery
High-risk tumor	≥3 years of age with evidence of CSF spread (M1–M3) and/or those with less complete resection (≥1.5 cm^2^) or <3 years of age at diagnosis

## Histological Classification

**Figure 2:**
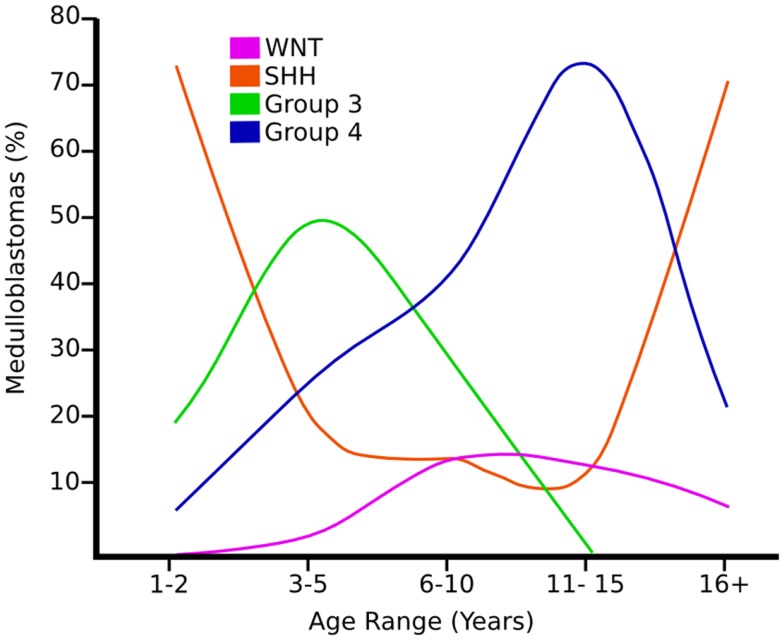
**Graph showing the age distribution for different subgroups of medulloblastoma adapted from Ref. (8)**.

The World Health Organisation (WHO) classification system 2007 for medulloblastomas uses histology to classify medulloblastomas, which can be considered three major groups including the classic subtype; desmoplastic/nodular/medulloblastoma with extensive nodularity (MBEN) subtypes; and large cell/anaplastic medulloblastoma subtypes (Figures 1 and 2). Classic medulloblastoma represents the most common histological subtype (66%) (1), and is composed of sheets of densely packed small round blue cells (basophilic) with a high nuclear to cytoplasmic ratio, mitotic and apoptotic activity, and may occur in the midline (1).

Desmoplastic/nodular medulloblastomas/MBEN (15%) typically carry a favorable prognosis, and may arise laterally in a cerebellar hemisphere (1, 6). Desmoplastic medulloblastomas also comprise small round blue tumor cells, but typically harbor reticulin-free “pale islands” within a reticulin-rich stroma, which are often immunopositive for synaptophysin indicating neuronal differentiation (1, 4, 6). Anaplastic medulloblastomas (15%) are characterized by marked nuclear pleomorphism, nuclear molding, and cell–cell wrapping (1) and the large cell variant (2–4%) displays a monomorphous population of large cells whose nuclei exhibit prominent nucleoli (1, 7). Both variants are characterized by a very high proliferative activity, abundant apoptosis, and a much poorer prognosis (1, 11, 12).

The majority of medulloblastomas exhibit neuronal differentiation in the form of immunoreactivity to synaptophysin and some also display focal glial differentiation (Glial fibrillary acidic protein (GFAP) immunopositivity) (1, 7, 8). Rare examples show myogenic differentiation (medullomyoblastoma) or melanotic differentiation (1, 7, 8).

## Molecular Subgroups

**Figure 3:**
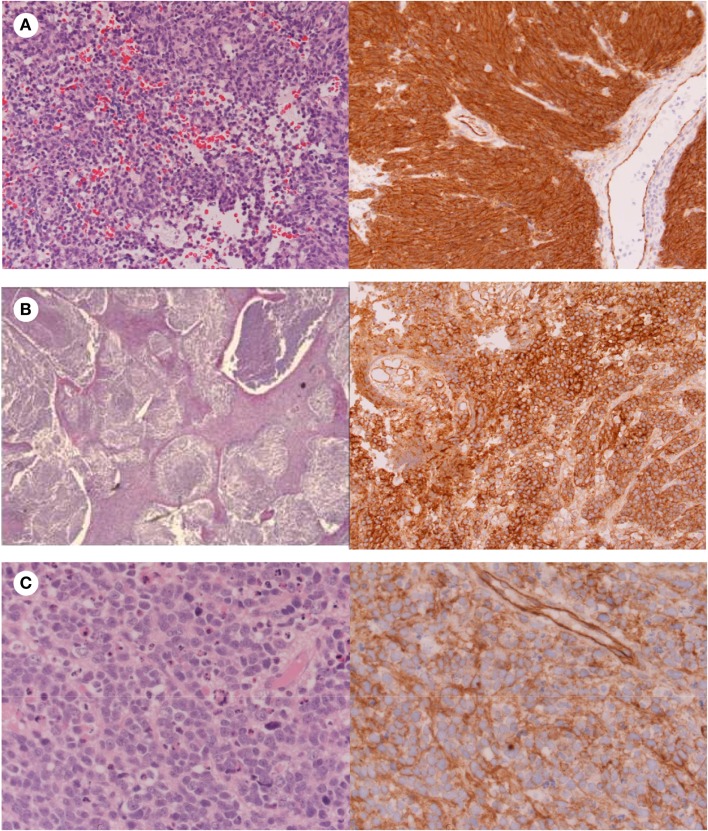
**Histology showing (A) Classic medulloblastoma with nuclear β-catenin immunostaining; (B) Nodular medulloblastoma with cytoplasmic β-catenin immunostaining; (C) Anaplastic medulloblastoma with cytoplasmic β-catenin immunostaining**.

More recently a consensus conference in Boston in 2010 supported classification of four main subgroups of medulloblastomas based on the molecular profiling (7, 8, 13–17) (Figure 3). The Wnt and Shh groups were named after the predominant signaling pathways thought to be affected in their pathogenesis. Less is known currently regarding the pathogenesis of groups 3 (tending to harbor *MYC* amplification) and 4 (tending to have isochromosome 17q) and therefore generic names were chosen until it is better understood (8). The Shh group has become of increasing interest because of the availability and temporary success of small molecule inhibitors to smoothened (SMO), which is part of the Shh pathway. All four groups show relatively distinct variation in demographics, histology, genetic profile, and clinical outcome (8). For a detailed comprehensive review on the molecular subgroups of medulloblastoma, see the consensus paper by Taylor et al. (5).

## Wnt Medulloblastomas

**Figure 4:**
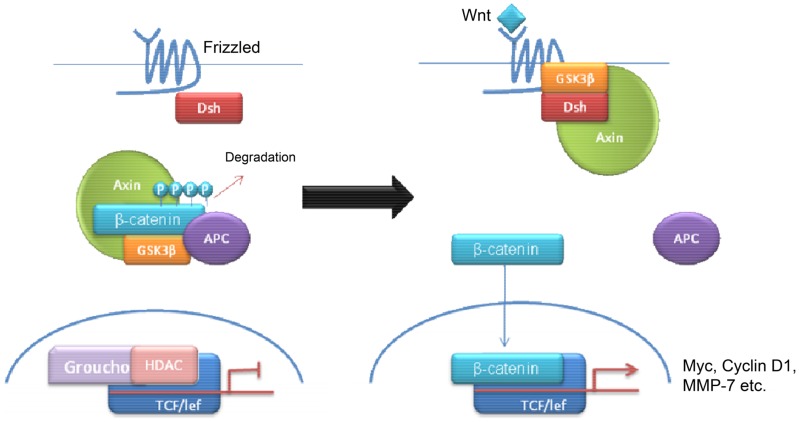
**Schematic overviewing Wnt signaling**.

Wnt tumors are thought to be the rarest subgroup of medulloblastoma, accounting for 11% (9), but they have probably been the most studied and have a very good long-term prognosis with overall survivals reaching 90% (18) (Figure 4). Wnt tumors also show a specific age distribution being almost absent in infants (aged <4 years) (see Figure 2) but predominantly affecting children with a peak incidence of 10–12 years (see Figure 2) (9). Wingless (Wnt) is a family of growth factor receptors that are involved in embryogenesis and also in cell–cell control mechanisms (9). Wnt tumors are thought to arise from mossy-fiber neuron precursors, which may be involved in the formation of synapses in the developing cerebellum (19). The majority of Wnt medulloblastomas show classic histology, however rarely, they are phenotypically large cell/anaplastic (1) and may remarkably retain their relatively good prognosis with this phenotype (14). Molecular analysis of sporadic Wnt medulloblastomas commonly shows CTNNB1 mutations, which encode β-catenin (see Figure 1) (14). Moreover, germline mutations of the Wnt pathway inhibitor APC predispose to Turcot syndrome in which medulloblastomas may occur (7). Other less common mutations are found in sporadic medulloblastomas, including APC, AXIN1, and AXIN2, which are also keys to this pathway (14). A recent paper has also identified mutations in the RNA helicase DDX3X, which potentiates transactivation of a TCF promoter, which is further downstream (15). Most mutations result in over-activation of the Wnt signaling pathway with increased nuclear (as opposed to cytoplasmic) immunohistochemical staining for β-catenin, which can be relatively easily identified by neuropathologists (15). Stimulation of Wnt signaling results in nuclear accumulation of β-catenin which complexes to TCF-4/lef-1 and functions in cell division and proliferation (transcribes c-myc and cyclin D1), breakdown of the extracellular matrix, as well as cell–cell adhesion (20). Interaction between the PI3K/Akt and Wnt pathways occurs in medulloblastomas and this appears to be crucial for tumor survival (20).

Wnt medulloblastomas appear to be associated with the loss of chromosome 6 and interestingly, they rarely express chromosome 17 aberrations which are the most common chromosomal alterations detected in other medulloblastoma subgroups, particularly groups 3 and 4 (20). Wnt medulloblastomas also have high levels of expression of *MYC* (5). A recent paper showed that mutations in CTNNB1 disrupt the normal differentiation and migration of progenitor cells on the dorsal brainstem, resulting in the accumulation of aberrant cell collections, which may relate to their midline origins (19).

## Shh Medulloblastomas

**Figure 5:**
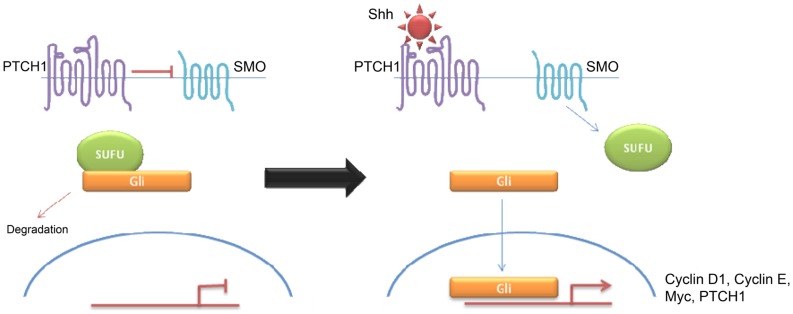
**Schematic overviewing Shh signaling**.

Shh tumors are thought to account for 28% of all medulloblastomas (7) (Figure 5). They have an intermediate prognosis between good prognosis Wnt tumors and poor prognosis group 3 tumors, and may be similar in prognosis to group 4 (5, 20). Shh medulloblastomas show a dichotomous age distribution being more common in both infants ( <4 years) and adults ( >16 years) (see Figure 2) (20). Aberrant Shh signaling in normal human development can cause holoprosencephaly, a disorder which affects the midline of the face and nervous system, and there is an increased risk of infant medulloblastoma in Gorlin syndrome, which have germline mutations in PTCH, the Shh receptors (21). The sonic hedgehog (Shh) pathway plays a key role in normal cerebellar development where it induces proliferation of neuronal precursor cells in the developing cerebellum and other tissues (22). The Shh ligand is normally secreted by Purkinje neurons and promotes formation of the external germinal layer from the upper rhombic lip (21), and Shh tumors are thought to arise from the granule neuron precursor cells (23).

Desmoplastic/nodular and MBEN are almost exclusively associated with Shh pathway activation, although pathway activation is also observed in classic and large cell/anaplastic tumors (24, 25). Molecular analysis of sporadic medulloblastomas commonly shows Patched-1 (PTCH1) mutations, although mutations in SMO and Suppressor of Fused (SUFU) have been described (23–25). All mutations result in over-activation of the Shh signaling pathway. Binding of Shh to its receptor PTCH1 relieves tonic inhibition of SMO and allows release of the Gli family of transcription factors from inhibitory protein complexes, such as SUFU (26). Activation of the hedgehog pathway leads to an increase in Snail protein expression and a resultant decrease in cell–cell adhesion (26). Hedgehog signaling also appears to be a crucial regulator of angiogenesis and thus metastasis (27). Interestingly, there seems to be some overlap between Wnt and Shh signaling indicating that there may be a common therapeutic target (26, 28–31). Shh have high levels of expression of *MYCN* (5) Similarly, both of these subtypes show over-expression of genes involved in Notch and Platelet-derived Growth Factor (PDGF) signaling (9). Shh medulloblastomas appear to be almost exclusively associated with deletions of chromosome 9q, which is also the location of the PTCH1 gene (9q22) (8). While Shh tumors have been largely identified using transcriptional profiling, immunohistochemistry using, SFRP1, GLI1, and GAB1 have been proposed (5, 18).

## Group 3 Medulloblastomas

Group 3 tumors account for 28% of all medulloblastomas, and conceptually it may be convenient to consider them as being associated with *MYC* amplification (not *MYCN)* but not exclusively (5, 7, 16, 32). They are currently detected by transcriptional profiling, although immunohistochemistry for NPR3 has been proposed (7, 16, 32). They are associated with the worst prognosis of all the subgroups and are frequently metastatic (8). Group 3 tumors are found in infants and children but very rarely in adults (see Figure 2) (5). Similar to group 4 tumors, relatively little is known about the molecular pathogenesis of group 3 tumors and they are grouped according to similar transcription profiles (7). Group 3 medulloblastomas are mostly classic or large cell/anaplastic morphology (5). *MYC* amplification appears to be highly associated with group 3 tumors and is associated with a worse prognosis (14). It has been proposed that group 3 medulloblastomas are further categorized in to 3α and 3β based on the expression of Myc (5) with 3α tumors have *MYC* amplification and hence carry a worse prognosis (5) and 3β not over-expressing *MYC* and have a similar prognosis to group 4 tumors – underlying the fluid nature of these classifications at present (5). There is a possible role of the developmentally regulated transcription factor OTX2 in the development of groups 3 and 4 medulloblastomas (33). Interestingly, OTX2 had been shown to transcriptionally up-regulate the oncogene Myc (33). 26% of group 3 tumors have isochromosome 17q, however, group 3 tumors are much more likely than group 4 tumors to show gain of chromosome 1q and/or loss of chromosome 5q and chromosome 10q (5).

## Group 4 Medulloblastomas

Group 4 medulloblastomas are thought to be the most common “typical” subgroup of medulloblastoma, accounting for around 34% (5), and can be thought of conceptually as being associated with isochromosome 17q (5). Group 4 medulloblastomas rarely affect infants (0–3 years) and mainly affect children, with a peak age of 10 years (see Figure 2) (5). They are also currently detected by transcriptional profiling, although immunohistochemistry for KCNA has been proposed and is awaiting validation (5).

Although they frequently metastasize, they still have an intermediate prognosis, compared with the poor prognosis of group 3 (4, 13, 16, 18). The vast majority of group 4 medulloblastomas have a classic histology, although less frequently they can have a large cell/anaplastic morphology (5). Almost two-thirds of group 4 medulloblastomas have an isochromosome 17q (i17q) though occasionally isolated 17p deletions are seen (7, 8). Isochromosome 17q and 17p mutations are also observed in some group 3 medulloblastomas though less frequently (5). Group 4 medulloblastomas are associated with CDK6 and *MYCN* amplification but minimal *MYC* over-expression (8). Interestingly, chromosome X loss is seen in 80% of females with group 4 medulloblastomas (8) Groups 3 and 4 medulloblastomas have recently been shown to have EZH2 and KDM6A alterations which are involved in histone methylation (specifically H3K27) (34, 35).

Other histone methylases/acteylases, such as HDAC5, HDAC9, MLL2, and MLL3, have also been found to be over-expressed in medulloblastomas, but in these studies the authors did not investigate their prevalence in individual subgroups (34, 36). Interestingly, the HDAC5 gene locus is located on chromosome 17q, which is commonly amplified in group 4 tumors (34, 36).

## Therapeutic Targeting of Medulloblastomas

Current treatment strategies for medulloblastoma are developed based on the risk stratification and age of the patient (see Table 1). In all subgroups of patients, surgery is first line treatment, which aims for maximal tumor resection. Postsurgical treatment is then varied with high-risk groups receiving higher-dose multimodal chemotherapy protocols in addition to craniospinal radiation (2, 37–40). There are substantial concerns, however, over the long-term neurocognitive sequelae of whole brain radiation on the developing brain meaning that in patients younger than the age of 3 years (or sometimes as old as 7 years) craniospinal radiation is often delayed or eliminated (3, 38–44). Many trials are currently being undertaken, which are aimed at optimizing the doses and drugs used in chemotherapy regimes in children to achieve maximum effect, however, these will not be covered in this review.

### Targeting the Wnt pathway

In contrast to the Shh pathway, relatively few drugs have been developed, which specifically target the Wnt pathway. The reason for this may be the inherent challenges of targeting the Wnt pathway (20, 45–47). While the Frizzled receptor would make a possible target, the majority of mutations in medulloblastomas occur downstream of this by mutations in CTNNB1, which encodes for β-catenin (20, 48, 49). Recently, a group found a naturally occurring compound in beetles termed cantharidin (derivative norcantharidin) which blocked Wnt signaling *in vitro* and reduced the size of intracranial tumors in a mouse model *in vivo* (50). Cantharidin and norcantharidin are known to inhibit protein phosphatases 1 and 2A (PP1 and PP2A) (51). It is thought that PP2A is required for Wnt mediated β-catenin stabilization downstream of the Wnt ligand (51). This represents a possible model for the development of synthetic derivatives although more research is needed. This may also represent a possible treatment for other types of medulloblastoma because activated Wnt signals interact with other signaling pathways (20, 26, 45, 49).

### Targeting the sonic hedgehog pathway

Since the discovery of a naturally occurring hedgehog pathway inhibitor cyclopamine, a number of cyclopamine derivatives have been developed with increased potency and bioavailability (22, 52–57). Similar to cyclopamine, these drugs act by inhibition of SMO (23, 57, 58). The most studied of these analogs is Vismodegib (GDC-0449), however, much of the research have been conducted in basal cell carcinomas (BCCs) rather than medulloblastomas because an increased prevalence of BCCs and a similar molecular pathogenesis (23). Unfortunately, a number of tumors treated with Vismodegib later acquired resistance by *de novo* mutations, specifically D473H point mutations (23). A new drug, Saridegib (IPI-926), showed decreased drug resistance in mouse models, and may be of future interest (28). Other SMO inhibitors have been developed but many of these have not entered clinical trials (23–25, 28, 29, 57–61).

Interestingly, new *in vitro* studies have highlighted a role for arsenic compounds in the treatment of hedgehog-driven cancers through a different method to other drugs (29). Arsenic compounds appear to disrupt tumorigenesis by targeting GLI, which are hedgehog signaling pathway components downstream of SMO and PTCH1 and so may be useful in tumors resistant to treatment with SMO inhibitors (29). Two other drugs, HPI 1 and 4, have also been found to inhibit proliferation of cerebellar granule neuron precursors, which expressed an oncogenic form of SMO that was resistant to cyclopamine (57).

### Targeting groups 3 and 4 medulloblastomas

Owing to the relative paucity of information on the underlying pathophysiology of groups 3 and 4 medulloblastomas, no specific treatments have been developed to target them as yet. The histone methylases EZH2 and KDM6A represent possible future targets of these subgroups as they appear to be exclusively expressed in groups 3 and 4 medulloblastomas (34, 35, 61). Similarly, there may be a role for histone deacetylase inhibitors, considering the possible amplification of HDAC5 in association with i17q (35). Some preclinical studies with histone deacetylase inhibitors have already shown some promise in medulloblastomas *in vitro*, however, these were not associated with molecular profiling of the tumors and identification of subgroups (35).

## Conclusion

The identification of different molecular pathways involved in the pathogenesis of medulloblastomas provides exciting new therapeutic targets for the development of new drugs with reduced side effects (62–70). Although this classification provides a simplified molecular schema for subdividing tumors, it does not take into account emerging work around molecular heterogeneity within tumors, which may become increasingly important as different molecular therapies enter clinical use. Moreover, in the future a systems biology approach may be relevant when considering these complex and intercommunicating pathways.

## Conflict of Interest Statement

The authors declare that the research was conducted in the absence of any commercial or financial relationships that could be construed as a potential conflict of interest.
